# Diurnal Variation of Essential of the Oil Components of Pycnocycla spinosa Decne. ex Boiss

**DOI:** 10.17795/jjnpp-12229

**Published:** 2014-02-20

**Authors:** Gholamreza Asghari, Houshfar Gholamali, Zahra Mahmoudi, Matin Asghari

**Affiliations:** 1 Isfahan Pharmaceutical Sciences Research Center, Isfahan University of Medical Sciences, Isfahan, IR Iran; 2 Department of Medicinal Chemistry, Faculty of Pharmacy and Pharmaceutical Sciences, Isfahan University of Medical Sciences, Isfahan, IR Iran; 3 Department of Agricultural Biotechnology, Tehran University, Tehran, IR Iran

**Keywords:** Oils, Volatile, Plants, Gas Chromatography-Mass Spectrometry

## Abstract

**Background:**

*Pycnocycla spinosa* Decne. ex Boiss is an aromatic plant which showed relaxant effects on isolated ileum contractions and antidiarrheal activity. Thirty four components have been extracted from *P. spinosa* essential oil, of which several major constituents were found to show seasonal variation.

**Objectives:**

The aim of this work is to evaluate the diurnal variation of its oil constituents during specific hours of the day.

**Materials and Methods:**

The *Pycnocycla spinosa samples* were collected at different times of the day. The hydro-distilled aerial parts oils of collected *P. spinosa* were analyzed by GC and GC/MS.

**Results:**

Fourteen monoterpenoid and nine sesquiterpenoid components were identified, of which the fluctuating constituents were *p*-cymene, *trans*-β-ocimene, β-citronellol, citronellyl pentanoate, geranyl isovalerate, α-humulene, caryophyllen oxide, α-cadinol, and α-eudesmol. The content of *p*-cymene in the essential oil in different daily times varied from 0.16 to 4.19%, and the geranyl isovalerate 7.75 -23.99%.

**Conclusions:**

Essential oils with different qualities can be obtained according to the harvest time of the plant in a day.

## 1. Background

*Pycnocycla spinosa* Decne. ex Boiss is a variety of *Pycnocycla* which belongs to Umbelliferae. It is an aromatic plant distributed in central parts of Iran (1). It was reported that the essential oil and extract of *P. spinosa* have relaxant effects on isolated ileum contractions in relatively low concentration. The essential oil has more potent ileum relaxant effects than its extract (2, 3). In addition, comparative studies have been shown that the antidiarrheal activity of *P. spinosa* extract and its effect on small intestinal transition of charcoal meal are relatively similar to loperamide (4). The hydrodistilled aerial parts oil of *Pycnocycla spinosa* was analyzed and thirty-four components have been identified. The most abundant constituents identified in the essential oil are geranyl isopantanoate, caryophyllene oxide, β-eudesmol, citronellol, elemicin, *p*-cymene, citronellyl acetate, α-cadinol, nonadecane, sabinene, octanal, δ-candinene, methyl eugenol, decanal, *trans*-β-ocimene, limonene, *trans*-caryophyllene and octadecane respectively, of which several major constituents were found to show seasonal variation (5, 6). There are no data concerning the diurnal variation of these constituents during specific hours of the day. It is well known that the concentration of phytochemicals varies during the day. Diurnal variation in plant secondary chemical components has been observed, either related to light conditions (7, 8), water deficit (9) or more complex interactions (10). Diurnal cycles characterize the accumulation of many compounds in plants. This is an expression of changes in enzymatic activities in the daily courses; such variations are suggested to be probably caused by variations in temperature or light conditions. This paper reports the diurnal variation of the essential oil from aerial parts of the plant. There has been no research regarding this issue.

The amount of a compound is usually not constant throughout the life of a plant. The stage at which a plant is collected is, therefore, very important for maximizing the yield of the desired constituents. It is well known that the concentration of many metabolites in plants varies during the day and season (11-13). Diurnal fluctuations of the alkaloid concentration in the *Lupinus* spp have previously been explained (14). The yield and composition of the essential oil from the leaves of Eucalypt (*Eucalyptus nicholii*), Rosemary (*Rosmarinus officinalis* L.), White Cedar (*Thuja occidentalis* L.) and Lawson Cypress (*Chamaecyparis lawsoniana*) was reported to have diurnal variation (15). However, a limited published data is available for the role of the time harvesting on chemical composition of essential oil (16-24).

## 2. Objectives

The essential oils composition of *Pycnocycla spinosa* was found to show seasonal variation (6). There are no data concerning the diurnal variation of its oil constituents during specific hours of the day. In this study, the diurnal variation was investigated.

## 3. Materials and Methods

### 3.1. Plant Materials

*Pycnocycla spinosa* var. spinosa was collected from Isfahan University campus and was identified by the botanist Mr. Mehregan in department of biology at Isfahan University. A voucher specimen (A24) was authenticated and then deposited in the herbarium of Faculty of Pharmacy and Pharmaceutical Sciences, Isfahan, Iran. Samples of wild *P. spinosa* were collected in the middle of the flowering period. The samples were harvested every three-hour over a 12-hour period from 7:00 AM to 7:00 PM.

### 3.2. GC/MS Analysis

The hydrodistilled aerial parts oils of *P. spinosa* were analyzed by GC and GC/MS. Gas chromatography analysis was carried out using Perkin-Elmer 8500 gas chromatograph with flame ionization detector (FID) detector and a BP-1 capillary column (39 m × 0.25 mm; film thickness 0.25 µm). The carrier gas was helium with a flow rate of 2 mL/min, the oven temperature for the first 4 minutes kept at 60˚C and then increased at 4˚C/min until reached to the temperature of 280˚C, injector and detector temperatures were set at 280˚C. Confirmation of peak identity was effected by co-chromatography with standards and GC-MS. The mass spectra were recorded on a Hewlett Packard 6890 MS detector coupled with Hewlett Packard 6890 gas chromatograph equipped with HP-5MS capillary column (30 m × 0.25 mm; film thickness 0.25 µm). The gas chromatography condition was as mentioned previously. Mass spectrometer condition was as follow: ionized potential 70 eV, source temperature at 200˚C. Identification was based on retention data and computer matching with the Wiley 275.L library as well as by comparison of electron-impact-mass spectra (EI-MS) with those relevant reference samples and the literature (25, 26).

## 4. Results

**Figure 1.**
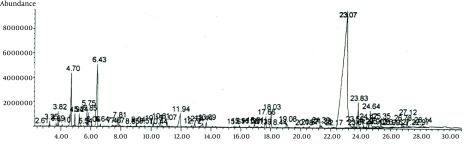
GC Chromatogram of *P. spinosa* Essential Oil

The hydrodistilled aerial parts oil of *Pycnocycla spinosa*, collected on different time of day, was analyzed and thirty-four components identified, of which several constituents were found with diurnal variation. The GC chromatogram of *P. spinosa *essential oil is presented in Figure 1 and diurnal variations of fluctuating monoterpenoid and sesquiterpenoid components are shown in Table 1. 

**Table 1.** The Diurnal Variation of Fluctuating Constituents in the Essential oil of *P. Spinosa *^a ^

Compounds	7:00 AM	10:00 AM	1:00 PM	4:00 PM	7:00 PM
***P*****-cymene**	2.94	0.16	1.40	1.56	4.19
**t-β-ocimene**	1.21	1.49	2.19	0.11	0.00
**β-citronellol**	7.26	5.01	5.67	8.11	1.55
**Citronellyl Pentanoate**	10.32	14.31	7.31	18.17	10.53
**Geranyl isovalerate**	17.91	19.56	7.75	11.61	23.99
**α-humulene**	1.32	1.11	0.00	0.46	0.57
**Caryophyllen oxide**	3.99	0.00	0.00	0.0	4.60
**α-cadinol**	2.86	0.59	0.00	2.12	3.01
**α-eudesmol**	5.72	6.46	3.46	4.11	5.12

^a^ % in Oil, average of triplicate

As presented in Table 1, the fluctuating monoterpenoid constituents were *p*-cymene, *trans*-β-ocimene, β-citronellol, citronellyl pentanoate, and geranyl isovalerate. Also four fluctuating sesquiterpenoid constituents were α-humulene, caryophyllen oxide, α-cadinol, and α-eudesmol (see Table 1). Several studies on aromatic plants have shown that the essential oil composition may vary considerably throughout a year (27, 28). The results showed that the content of *p*-cymene in the essential oil in different daily times 0.16 - 4.19% varied, and of geranyl isovalerate did 7.75 - 23.99% (see Table 1). Seasonal variation has also been noticed for *p*-cymene in the essential oil of *Origanum onites* (29). Results of diurnal variation of β-citronellol and citronellyl pentanoate were correlated. β-citronellol and citronellyl pentanoate reached to their lowest level at 7:00 and 1:00 PM. respectively, while both components reached to their highest levels at 4:00 PM. Similarly, it was reported that the percentage of citronellol, main components of rose oil, increased with delay in harvesting. Geraniol content of rose oil was maximum when the flowers were harvested at 10:00 AM, but after that there was significant reduction in its concentration up to 06:00 PM (24). As illustrated in Table 1, there is severing drop in citronellyl pentanoate content at 1:00 PM during day period. It may indicate some correlation between β-citronellol content and the level of its pentanoate ester. Results of diurnal variation of sesquiterpenes, α-humulene, caryophyllen oxide, α-cadinol, and α-eudesmol are presented in Table 1. Seasonal and diurnal variation has also been noticed for *Hymenaea courbaril*, *Copaifera officinalis*, and *Copaifera pubiflora*, which content of sesquiterpenes varies greatly during the year (30). α-cadinol showed the highest concentration at 7:00 PM. Also plant harvested early in the morning at 7:00 a.m. and late evening at 7:00 PM. provided essential oil with a high caryophyllen oxide content. Seasonal variation also was observed for this compounds (5). These variations were probably are related to the temperature or light variations. Plant cells are dependent on light for their growth and development. Light is also important for the production of metabolites by plant cells, and so also are irradiance, wavelength, and exposure time. As illustrated in Table 1, α-humulene reached to its highest level at 7:00 AM, while the lowest level was observed at 1:00 PM. In contrast, this compound was reported to be at highest level at 2:0 PM in Rosa damascene essential oil (31). It seems its fluctuation may not be related to the temperature or light but probably to genus and genetics of the plant. Caryophyllen oxide and α-cadinol reached their highest level at 7:00 PM. The highest content of eudesmol was observed when the plant was harvested at 10:00 AM. Variation in the other mono- and sesquiterpenes due to time were not significant. 

## 5. Discussion

The interesting point to note is that diurnal cycles characterize the accumulation of 5 monoterpenes and 4 sesquiterpenes of thirty-four components identified in the essential oils of *P. spinosa*. Such variation has been reported in many compounds of the plants (32, 33). It seems that this is an expression of changes in enzymatic activities; such variations are suggested to be probably caused by variations in temperature or light conditions in different courses of the day (34). Light effects on the accumulation of secondary metabolites may occur on three different levels; through direct control of product concentrations, influences on membrane permeability, and influence on enzymatic reactions. The latter may occur either by varying enzyme activities or altering the concentrations of the involved enzymes. In conclusion, variations in essential oil components of *P. spinosa* in response to diurnal changes may indicate that essential oil with different qualities can be obtained according to the harvest season and harvest time of the plant.
